# Perceived duration of brief visual events is mediated by timing mechanisms at the global stages of visual processing

**DOI:** 10.1098/rsos.160928

**Published:** 2017-03-01

**Authors:** Lee Beattie, William Curran, Christopher P. Benton, Julie M. Harris, Paul B. Hibbard

**Affiliations:** 1 School of Psychology, Queen's University Belfast, Belfast, Northern Ireland, UK; 2 School of Experimental Psychology, University of Bristol, Bristol, Avon, UK; 3 School of Psychology and Neuroscience, University of St Andrews, St Andrews, Fife, UK; 4 Department of Psychology, University of Essex, Colchester, Essex, UK

**Keywords:** adaptation, motion processing, time perception

## Abstract

There is a growing body of evidence pointing to the existence of modality-specific timing mechanisms for encoding sub-second durations. For example, the duration compression effect describes how prior adaptation to a dynamic visual stimulus results in participants underestimating the duration of a sub-second test stimulus when it is presented at the adapted location. There is substantial evidence for the existence of both cortical and pre-cortical visual timing mechanisms; however, little is known about where in the processing hierarchy the cortical mechanisms are likely to be located. We carried out a series of experiments to determine whether or not timing mechanisms are to be found at the global processing level. We had participants adapt to random dot patterns that varied in their motion coherence, thus allowing us to probe the visual system at the level of motion integration. Our first experiment revealed a positive linear relationship between the motion coherence level of the adaptor stimulus and duration compression magnitude. However, increasing the motion coherence level in a stimulus also results in an increase in global speed. To test whether duration compression effects were driven by global speed or global motion, we repeated the experiment, but kept global speed fixed while varying motion coherence levels. The duration compression persisted, but the linear relationship with motion coherence was absent, suggesting that the effect was driven by adapting global speed mechanisms. Our results support previous claims that visual timing mechanisms persist at the level of global processing.

## 1. Introduction

Picture a cloud of starlings coming together in a murmuration at dusk before roosting. The starlings fly in close formation with sudden changes in flight direction, yet they do not collide with one another. Such aerodynamic feats require exquisite timing of sensorimotor processing in the sub-second range. Likewise, everyday human activities, from holding conversations, to driving in fast-moving traffic, depend on our ability to perceive the duration and temporal order of brief events around us. This ability to accurately encode the duration of sub-second events is crucial to our survival and interaction with our environment. There is a growing body of evidence that sub-second event duration is encoded by modality-specific timing mechanisms [1–4]. In the case of the visual system, Johnston *et al.*'s research [5] points to the involvement of spatially localized temporal mechanisms in encoding the duration of brief visual events. Johnston *et al.* found that prior adaptation to an oscillating, high temporal frequency sine wave grating resulted in participants underestimating the duration of a 600 ms test stimulus drifting at a medium frequency and presented at the same location as the adaptor. This so-called duration compression effect is highly tuned to the spatial location of the adaptor [6], suggesting that, in the case of the visual system, sub-second duration encoding is mediated by the activity of multiple temporal mechanisms.

Since Johnston *et al*.'s initial report of the duration compression phenomenon, there has been ongoing debate about the characteristics of the underlying timing mechanisms. Research has focused on three related questions: the frame of reference (retinotopic or spatiotopic) within which the mechanisms operate; their location (pre-cortical or cortical) and whether the timing mechanisms occur at local or global stages of visual processing. In the case of the first question Burr *et al.* [7] report that, when changes in perceived speed of test stimuli are controlled for, duration compression occurs within a spatiotopic, but not a retinotopic, frame of reference. Given that visual area MT+ is the earliest visual area in which spatiotopic encoding is known to occur [8] and has been previously linked to sub-second timing [9,10], Morrone *et al.* [11] have identified MT+ as a candidate area for the location of the timing mechanisms underlying the duration compression effect. However, the spatiotopic view of duration compression has been challenged by evidence that timing mechanisms underlying the effect operate solely within a retinotopic frame of reference [12,13].

Latimer & Curran [14] noted that the contrasting results of previous experiments addressing the retinotopic–spatiotopic question may have been a consequence of employing stimuli that are not well suited to revealing spatiotopic mechanisms. They had participants adapt to a plaid stimulus whose component gratings drifted in directions ±70° either side of vertical upwards. Following adaptation participants judged the duration of a test random dot kinematogram (RDK) stimulus drifting in the same direction and at the same speed (3° s^−1^) as the adaptor plaid's pattern. It is known that drifting plaids selectively stimulate MT neurons tuned to the plaid's pattern motion direction [15]. Since MT direction-sensitive neurons respond to plaid pattern motion, and area MT+ is the earliest point in the motion pathway where spatiotopic encoding is known to occur, Latimer and Curran reasoned that the plaid adaptor and RDK test stimuli combination would be well suited to revealing spatiotopic mechanisms (if they exist) that contribute to the duration compression effect. They also reported that the duration compression effect occurred within both a retinotopic and a spatiotopic frame of reference. Thus, the research to date suggests that the timing mechanisms that encode the duration of brief visual events operate within both retinotopic and spatiotopic frames of reference.

Just as with the retinotopic–spatiotopic debate, there is disagreement on whether the timing mechanisms underlying the duration compression effect are cortical or pre-cortical. There is a large body of evidence to support both these positions, thus pointing to the possibility that timing mechanisms may exist at multiple levels of the visual system. For instance, evidence for pre-cortical timing mechanisms include reports that duration compression can also be induced by very narrow adaptors [6]; the effect is tightly tuned to the area of adaptation [6]; and the effect persists using adaptors that flicker above the flicker fusion level of V1 neurons but below the flicker fusion level of LGN neurons [16]. The existence of cortical timing mechanisms, on the other hand, is evidenced by reports that the duration compression effect is spatiotopically tuned [7,11,14], it undergoes interocular transfer [7] and it is direction contingent [13,17–19].

The third related issue concerning the duration compression effect, which is addressed in our experimental section, is whether the timing mechanisms underlying it are located at the local or global level of visual processing. Local processing occurs early in the visual hierarchy and is a consequence of neurons having small receptive fields; global processing, on the other hand, refers to processing further up the visual hierarchy where neurons with large receptive fields integrate information from local processing neurons. One way of addressing the local–global question is to use adaptor stimuli that are known to selectively stimulate neurons at either the local or global processing level. For instance, Johnston *et al*. [16] reported that the duration compression effect is induced following adaptation to an adaptor flickering at 60 Hz, a flicker rate which is detectable within the LGN but is practically invisible to cortical cells [20]. Since LGN neurons have small receptive fields, their apparent involvement in the duration compression effect points to duration encoding occurring at the local processing level. Curran & Benton [18] used plaid adaptors to probe whether visual timing mechanisms persist at the global processing level. They had participants adapt to a plaid comprising two sine wave gratings drifting ±70° either side of vertical upwards. Such plaid stimuli are known to selectively activate MT neurons tuned to the plaid's pattern motion direction [15]. Following adaptation, participants underestimated the duration of a random dot test pattern drifting at the same speed and in the same direction as the plaid pattern, suggesting that visual timing mechanisms also exist at the global processing level. Additional evidence for the existence of timing mechanisms at a global processing level is provided by a recent report that adaptation-induced duration distortion extends over an area approximately five times that of the adapting stimulus [21].

In this paper we describe a series of experiments in which we measure the extent to which the duration compression effect is driven by the adaptation of timing mechanisms at the global motion processing stage. In our first experiment we use motion coherence RDKs as adaptor stimuli in a duration adaptation experiment. Motion coherence is defined as the proportion of signal dots in an RDK that move in a given direction, with an increasing proportion of signal dots resulting in increasing motion coherence. It has been reported that macaque MT direction-selective neurons increase their firing rate in response to increasing motion coherence [22], and there is evidence that direction-selective neurons in the human brain exhibit similar response characteristics [23]. Thus motion coherence stimuli are well suited to uncovering timing mechanisms at a global processing stage that includes motion. Our initial results reveal a linear increase in duration compression magnitude as a function of adaptor stimulus motion coherence level. This result is consistent with the existence of timing mechanisms at the global processing stage. A follow-up experiment explored whether the changes in duration compression magnitude found in Experiment 1 were determined by the motion coherence level or global speed (which covaried with the coherence level) of the adapting stimulus. This was achieved by keeping the adaptor global speed fixed across a range of motion coherence levels. When the global speed was kept fixed, the duration compression effect persisted but, crucially, its magnitude did not vary with the motion coherence level.

## 2. Experiment 1

### 2.1. Participants

Five participants were tested in Experiment 1 and four were tested in Experiment 2, including two of the authors.

### 2.2. Apparatus and stimuli

Stimuli were random dot kinematograms, with equal numbers of black (0.9 cd m^−2^) and white (51.3 cd m^−2^) dots against a mean luminance (26.2 cd m^−2^) background, presented within a circular aperture (6.3° diameter). Adaptor and test stimuli were centred 5° left of fixation, and a comparison stimulus was centred 5° right of fixation. Stimuli were presented on a Sony GDM-F500R monitor driven by a Cambridge Research Systems VSG 2/5 graphics board at a frame rate of 120 Hz.

### 2.3. Procedure

**Figure 1.**
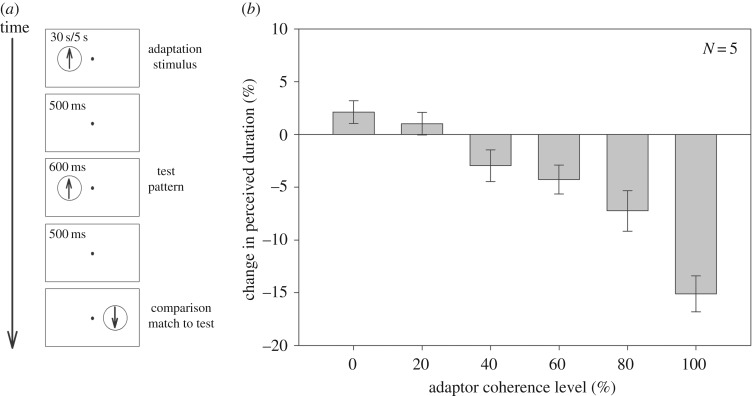
(*a*) Experimental timeline. (*b*) Change in perceived duration as a function of the adaptor coherence level. Negative and positive values indicate duration compression and duration expansion, respectively. It is clear that increasing the adaptor's motion coherence level leads to a concomitant increase in the magnitude of the duration compression effect (error bars are ±1 s.e.).

Observers adapted for 30 s to a moving random dot pattern centred 5° left of a central fixation point, in which each dot moved at the same speed 3° s^−1^ (figure 1*a*). Observers maintained fixation throughout each block of trials. Within each block of trials the adaptor pattern was assigned a fixed motion coherence value of 0%, 20%, 40%, 60%, 80% or 100%. The lifetime of each dot was set to the duration of the adaptor stimulus. In the case of 100% motion coherence all dots in the adaptor stimulus drifted upwards; for lower motion coherence levels the probability that a dot was assigned the signal motion direction (upwards) on each frame was determined by the motion coherence parameter. Thus, for the 20% motion coherence condition each dot had a 0.2 probability of being assigned the signal motion direction, and a 0.8 probability of being assigned a random motion direction, on a given frame. Using the terminology of Scase *et al.* [24], who looked at motion thresholds for a variety of different types of RDK, our random dot pattern is classified as random walk using the different rule. After 30 s adaptation, observers judged the duration of a brief (600 ms) upwards-moving (3° s^−1^), 100% coherence test pattern at the same location as the adapting pattern. Perceived duration of the test pattern was estimated by having observers judge whether a comparison pattern (100% coherence) of variable duration, centred 5° right of fixation and moving in the opposite direction to the test pattern, was of longer or shorter duration than the 600 ms test pattern.

To maintain adaptation, each subsequent test-comparison pair was preceded by a 5 s top-up presentation of the adapting stimulus. The presentation order of test and comparison stimuli was randomized on each trial. The comparison stimulus duration was chosen on each trial by an adaptive method of constants procedure [25] and was selected to optimize the estimation of the ‘point of subjective equality’ (PSE); that is when its duration appeared to equal that of the test stimulus. Each observer generated 24 PSEs—four for each motion coherence condition. Distortions in perceived duration of the test stimulus were calculated using the equation

(2.1) $$\frac{\text{PD}-\text{TD}}{\text{TD}}\times 100,$$

where TD is the test stimulus duration and PD is its perceived duration. Positive and negative distortion values reflect duration expansion and duration compression, respectively.

In this and the subsequent experiment, comparison stimulus speed was tailored specifically for each observer and matched to observers' perceived speed of the test stimulus, thus controlling for adaptation-induced speed distortions [26] and taking into account previous reports that perceived speed influences apparent duration [27,28]. The perceived speed measurements were gathered in an initial preliminary experiment that was identical to the duration experiment, with the exception that the test and comparison stimuli were presented for the same duration (600 ms), comparison speed varied from trial to trial and participants judged whether the comparison stimulus was moving faster or slower than the test stimulus.

### 2.4. Results

Figure 1*b* plots the duration distortion magnitude as a function of the motion coherence level of the adaptor stimulus. The data reveal a clear linear relationship between adaptor motion coherence and duration distortion magnitude—with increasing motion coherence resulting in greater duration compression (*F*_5,20_ = 13.88; *p* < 0.001).

Increasing motion coherence level results in increasing activity in area MT, an area thought to be responsible for global motion processing [29]. At first sight, therefore, our results are consistent with the duration compression effect being driven by adaptation of timing mechanisms at the global motion processing level. However, it has also been demonstrated that increasing motion coherence in this type of stimulus is accompanied by an increase in its perceived pattern speed, defined as the apparent speed of global motion in the signal direction [30,31]. It is possible that the observed change in duration compression may be a result of the speed at which the adaptor appeared to move in the signal direction, rather than its motion coherence. This alternative, perceived speed-based interpretation of our results is consistent with a previous report that the duration compression effect is speed-tuned [32]. Maximum compression occurs when the adaptor and test stimuli have the same drift speed; increasing the speed difference between adaptor and test stimuli results in the effect's magnitude reducing and eventually disappearing. Thus the increasing duration compression associated with increasing motion coherence in Experiment 1 may have nothing at all to do with motion coherence; rather it might simply be a consequence of the adaptor's global speed approaching that of the test stimulus. Our second experiment explored which of these two characteristics of the adaptor (pattern speed or motion coherence) was driving the change in duration compression.

## 3. Experiment 2: adaptor global speed, not coherence, determines duration compression magnitude

### 3.1. Procedure

In this experiment, we used adaptors that had one of five motion coherence levels: 40%, 60%, 70%, 80% or 100%. In contrast with Experiment 1, the *perceived* global speed of the adaptor was identical across all coherence levels, and was set to match the perceived global speed of the 70% coherence adaptor (perceived speeds were determined in a separate control experiment). Dot speed was 1.5° s^−1^ for the 70% coherence adaptor, resulting in a perceived global speed of approximately 1.05° s^−1^. As in Experiment 1, the comparison stimulus's speed was matched to observers' perceived speed of the test stimulus (actual test speed was 1.5° s^−1^). In all other respects, the procedure was identical to Experiment 1. If adaptor motion coherence underlies the increasing duration compression observed in the previous experiment, then we would expect to find the same linear relationship between the motion coherence level and the duration compression magnitude. If, on the other hand, the adaptor perceived pattern speed drives duration compression, there should be no change in compression magnitude. In the case of the latter, this would mean that coherence itself has no direct effect on duration.

### 3.2. Results

**Figure 2.**
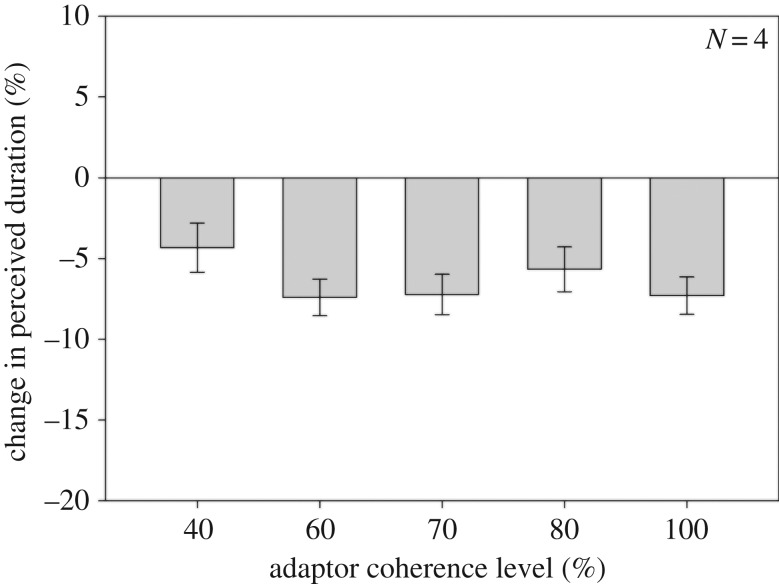
Results from Experiment 2, in which adaptor stimuli varied in their motion coherence level but had identical pattern speeds (see text for details). When the pattern speed is kept constant the apparent relationship between adaptor coherence and duration compression magnitude found in Experiment 1 is no longer evident.

While the results (figure 2) show a clear duration compression effect across all motion coherence conditions, there is no evidence for an effect of adaptor motion coherence on duration compression magnitude when the perceived global speed is held fixed (*F*_4,12_ = 0.61; *p* = 0.663). The results of Experiment 2, in conjunction with those of Experiment 1, therefore, suggest that duration encoding of the 600 ms test stimulus was mediated by differential adaptation of neural mechanisms that respond to the test pattern speed. The absence of a change in the duration compression magnitude in Experiment 2 can be explained by noting that the perceived pattern speed was identical across all adaptor coherence levels; given that the duration compression effect is known to be speed-tuned [32], the identical speeds of the adaptor stimuli would predict a similar duration compression magnitude across the different motion coherence conditions.

## 4. Discussion

Since its initial discovery [5], the duration compression effect has been used experimentally in attempts to reveal the characteristics of sub-second visual timing mechanisms. For example, there have been ongoing attempts to determine whether the neural timing mechanisms underlying the effect are cortical or pre-cortical in origins, whether they operate within a retinotopic or spatiotopic frame of reference, and whether they exist at local or global levels of motion processing. The experiments reported in this paper address the latter question. It may be the case that, as in the pre-cortical/cortical debate, both positions are correct and that visual timing mechanisms exist at both local and global processing levels. If this is the case, then demonstrating their existence would be best achieved using adaptor stimuli that selectively target timing mechanisms at either the local or global processing level [18].

In Experiment 1, we varied the motion coherence level of our adaptor stimulus in an attempt to selectively adapt timing mechanisms at the global processing level. Following adaptation, participants judged the duration of a 100% test stimulus at the adapted location. The results of Experiment 1 revealed a strong effect of the adaptor's motion coherence on the duration compression magnitude; specifically, the apparent duration compression magnitude of the test stimulus increased with increase in the motion coherence level of the adaptor stimulus. However, this apparently strong relationship between motion coherence level and duration compression magnitude was confounded by the observation that the perceived speed of stimulus motion covaried with its motion coherence level.

In Experiment 2, we varied the adaptor's motion coherence level while keeping its global speed identical across coherence level. If the results of Experiment 1 were a consequence of varying the adaptor's motion coherence level, then we would expect to find a similar linear relationship between motion coherence level and duration compression magnitude in Experiment 2. If, on the other hand, the changing duration compression magnitude in Experiment 1 was determined by the global speed of the adaptor, then we would anticipate that the duration compression magnitude should be constant across all coherence conditions of Experiment 2. We found the latter; keeping the adaptor's global speed constant while varying its motion coherence resulted in a similar duration compression magnitude across all coherence levels tested. This suggests that the increase in the duration compression magnitude observed in Experiment 1 was not a consequence of increasing the adaptor's motion coherence level, but was a consequence of the increasing pattern speed of the adaptor.

A reasonable conclusion to draw is that our results reflect a sensitivity to global motion speed of mechanisms mediating perceived duration. This conclusion would certainly fit well with the results of a study by Yamamoto & Miura [33] who studied perceived duration using plaids; they showed that perceived duration depends on the global pattern speed rather than the component speed. Of course, our study uses adaptation, and so is rather different from theirs. Our results suggest that it is the match in global speed between adaptor and test stimuli that drives duration compression; thus our results would seem to implicate area MT+ as a strong candidate site for the cortical adaptation driving our observed duration compression. This is because MT+ contains neurons with relatively large receptive fields [34], a characteristic which equips this area well for global motion processing [35–38], and up to 65% of MT neurons respond selectively to the pattern direction of random dot stimuli [39].

A recent report by Fornaciai *et al.* [40] also points to the existence of timing mechanisms at the global processing stage; but, intriguingly, the duration compression effect which revealed the mechanisms was found to be induced only by translational-motion adaptors, with other more complex motion adaptors having no effect on perceived duration. Since the adaptors used in our experiments contained dots moving in many different directions, it could be argued that duration compression is not restricted to just translational motion. However, we would suggest that our results align with those of Fornaciai *et al.*; this is because, despite containing dots with a wide range of trajectories, the perceived pattern motion of our adaptor stimuli is unidirectional translation. Indeed, this is consistent with the finding that the degree of duration compression was determined by the speed of global translational motion.

In conclusion, the results reported here add to the growing body of evidence that visual timing mechanisms exist at the global processing stages of the visual system, and that these cortical timing mechanisms are sensitive to global motion speed information.

## Data accessibility

Data and programming code for both experiments are available for download at http://dx.doi.org/10.5061/dryad.t6r83 [41].
